# Clinical Characteristics of Congenital Atresia of the Left Main Coronary Artery in 12 Children

**DOI:** 10.3389/fped.2022.866010

**Published:** 2022-04-29

**Authors:** Xiaokun Jiang, Wenqian Ye, Yanyan Xiao, Ling Han, Wenhong Ding, Wenxiu Li, Mei Jin, Xiaofang Wang, Qi Meng

**Affiliations:** Department of Pediatric Cardiology, Beijing Institute of Heart, Lung and Blood Vessel Diseases, Beijing Anzhen Hospital Affiliated to Capital Medical University, Beijing, China

**Keywords:** atresia of the left main coronary artery, congenital, children, coronary angiography, coronary angioplasty

## Abstract

**Background:**

Left main coronary artery atresia (LMCAA) is an extremely rare abnormality and only <100 cases have been reported worldwide. We describe the clinical manifestations, imaging features, prognosis, and treatments of LMCAA who were admitted in our department, which aimed to improve the clinical diagnosis and treatments of LMCAA in children.

**Methods:**

A retrospective study identified 12 patients diagnosed with congenital left coronary artery atresia at Pediatric Heart Center of Beijing Anzhen Hospital from June 2010 to June 2019. The clinical characteristics, imaging data, and treatment follow-up were analyzed.

**Results:**

Among the 12 cases, 8 were boys and 4 were girls; the age of onset was 2 months to 2 years old (median age 7 months); the age of diagnosis was 7 months to 6 years old (median age 2 years and 11 months). At the initial diagnosis, there were 4 cases of respiratory tract infection with cardiac murmur, 3 cases of cardiac shadow enlargement, 1 case of recurrent syncope, 2 cases of feeding difficulty with cardiac enlargement, and 2 cases of simple cardiac murmur. In 12 cases of electrocardiogram examination, 7 cases showed pathological Q waves of lead I, AVL and v4–v6; in 12 cases of chest X-ray examination, 8 cases showed cardiac shadow enlargement; in 12 cases of our hospital's first cardiac ultrasound examination, 4 cases were definitely diagnosed, and 8 cases showed the possibility of left coronary artery abnormality; in 5 cases of cardiac coronary CT angiography examination, 2 cases were confirmed, 2 cases reported suspected left coronary artery abnormality, and 1 case did not report abnormality; All cases were definitely diagnosed in 8 cases of angiography. Follow-up was performed from 1 to 8 years; one case died suddenly, one case of syncope after activity was treated by oral medication, 3 cases received open coronary angioplasty and mitral valvuloplasty, recovered well after operation, the rest of the children were treated by oral medication, and the symptoms are stable at present.

**Conclusions:**

Left main coronary artery atresia is difficult to diagnose and can result in heart failure early in life. Timely diagnosis and reasonable treatment are the keys to improve the prognosis.

## Introduction

The incidence of congenital coronary abnormalities was 0.6–1.3% in patients undergoing coronary angiography and 0.3% at autopsies (1). Among them, left main coronary artery atresia (LMCAA) is an extremely rare abnormality, and only <100 cases have been reported worldwide (2). LMCAA is characterized by the absence of left coronary ostium and the proximal left main trunk ends blindly, blood flows from the right coronary artery to the left *via* small collateral arteries. The symptoms of LMCAA can be different, and infants and children may be manifested as heart failure while some adults may be had no symptoms. The severity of symptoms is related to the degree of myocardial ischemia. This study retrospectively analyzed the clinical data of 12 children with congenital LMCAA referred to our department from June 2010 to June 2019, summarized the clinical features, imaging manifestations, treatments, and follow-up data of these children, to make early and correct diagnosis and to understand the prognosis of this disease.

## Methods

A retrospective study was performed to identify 12 patients diagnosed with congenital left coronary artery atresia at Pediatric Heart Center of Beijing Anzhen Hospital from June 2010 to June 2019. The patients met the following inclusion criteria: (1) The diagnosis was confirmed by cardiac coronary CT angiography (CTA) or coronary angiography in our hospital; (2) age of onset ≤ 14 years. Exclusion criteria were as follows: (1) left main coronary artery occlusion secondary to other diseases, such as Kawasaki disease, Takayasu arteritis, etc., and (2) complicated with other congenital heart malformations.

In this study, the relevant data and examinations that include medical history, laboratory examinations, electrocardiograms, and imaging examinations of the patients were collected by reviewing the cases. After a definite diagnosis, all the 12 children were given medicine therapy, and 3 of them underwent surgery. Regular outpatient follow-up was performed. All the children's guardians signed the informed consent. This study was approved by the hospital medical ethics committee.

## Results

Among the 12 patients, 8 were boys and 4 were girls. The onset age ranged from 2 months to 2 years (median age 7 months). The age of diagnosis was 7 months to 6 years (median age 2 years and 11 months). At the initial diagnosis, the manifestations were pneumonia, respiratory tract infection with cardiac murmur in 4 cases, cardiac shadow enlargement in 3 cases, recurrent syncope in 1 case, feeding difficulty with cardiac enlargement in 2 cases, and simple cardiac murmur in 2 cases. Among the 12 patients, the first visiting hospital was our hospital. Correct diagnosis was made in 1 case, and incorrect diagnosis was made in other 11 cases during their initial visit at other hospitals: 7 cases were misdiagnosed as valvular disease, of which 1 case was diagnosed as mitral valve prolapse and severe regurgitation who had received mitral valvuloplasty in other hospitals, 1 case was misdiagnosed as endocardial fibroelastosis, 1 case was misdiagnosed as left ventricular non-compaction, 1 case was misdiagnosed as dilated cardiomyopathy, and 1 case was misdiagnosed as the anomalous origin of the left coronary artery from pulmonary artery.

All the 12 patients underwent laboratory examination, electrocardiogram, chest radiography, and echocardiogram, five underwent cardiac CTA examinations, and 8 underwent coronary angiography.

**Figure 1:**
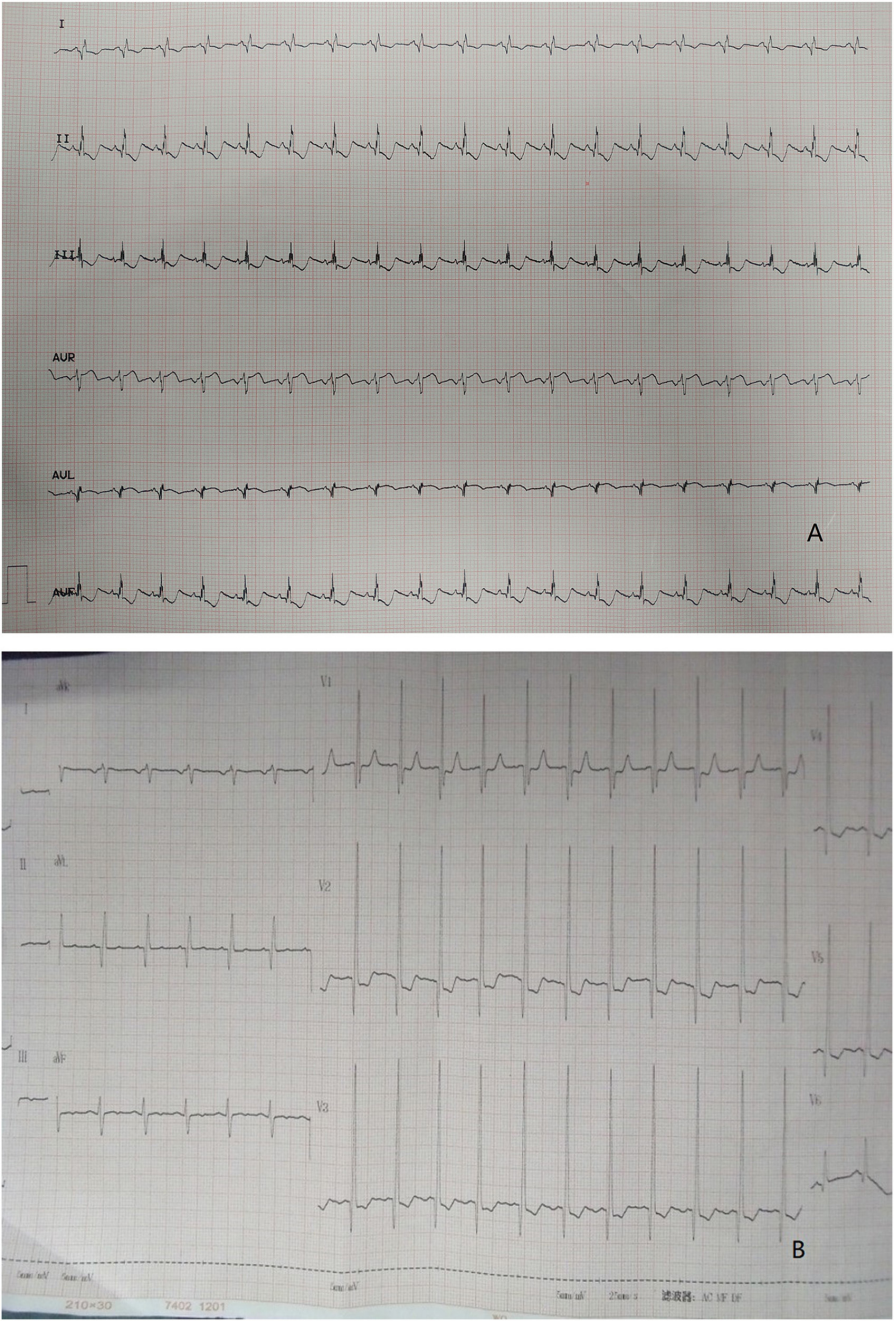
**(A)** ECG: pathological Q waves in lead I, AVL. **(B)** ECG: pathological Q waves in lead AVL and ST segment depression in lead V2, V3.

**Figure 2:**
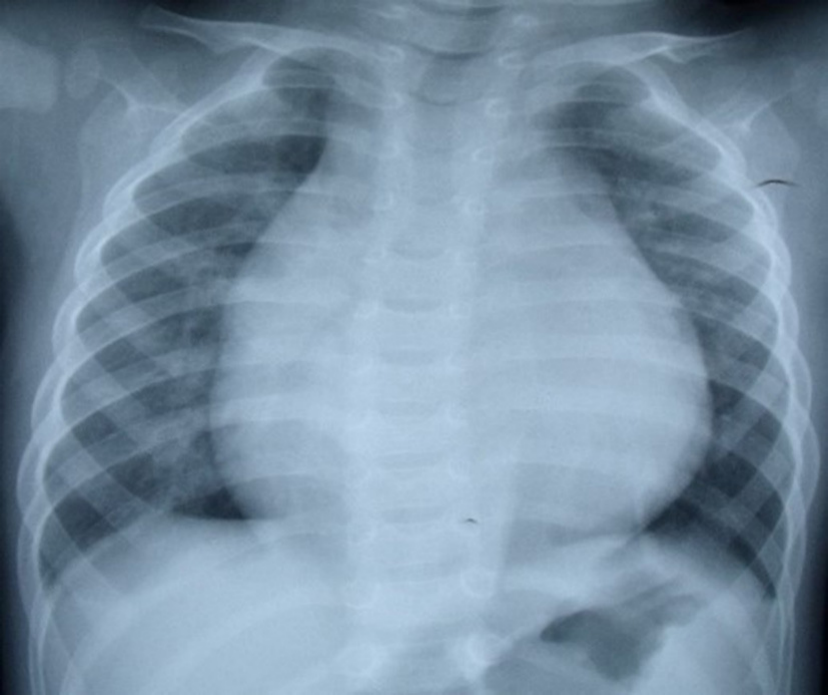
Chest X-ray:cardiac enlargement and cardiothoracic ratio 0.68.

In regard to laboratory examination, Creatine Kinase, MB Form (CK-MB) was abnormal in 2 cases. Brain natriuretic peptide (BNP) was slightly higher in 6 cases. Electrocardiogram was done in all patients. Then, 7 patients showed the presence of pathological Q waves and ST segment changes in lead I, AVL, and V4-V6 (Figure 1). The cardiothoracic ratio increased in 8 cases (≥0.55) (Figure 2).

**Figure 3:**
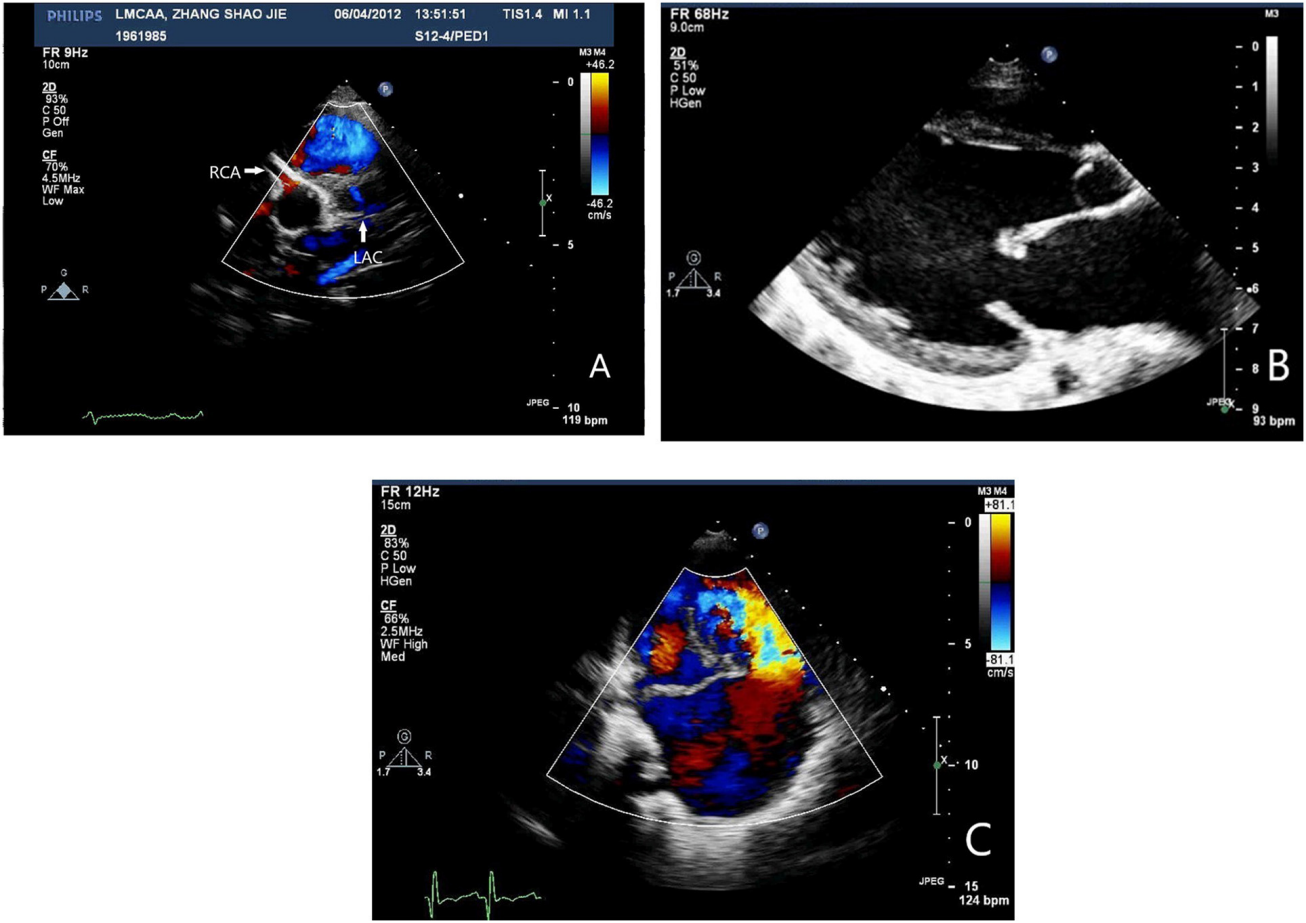
**(A)** Echocardiography: No left coronary artery opening in the left coronary sinus, the origin and course of RCA is normal. RCA dilated the signal of reverse perfusion blood flow can be seen in the slender left main coronary artery. **(B)** Echocardiography: Echo enhancement of mitral valve, papillary muscle, and endocardium. **(C)** Echocardiography: Massive mitral regurgitation.

A number of 4 cases diagnosed, and 8 cases suspected left coronary artery abnormality at the first-time echocardiogram was done in our hospital. Echocardiographic signs include the following: left ventricular enlargement in all 12 cases with *Z*-value of left ventricular end-diastolic diameter >2; ejection fraction of left ventricle decreased (<60%) in 3 cases (25%) and normal in 9 cases (75%); the origin of the right coronary artery is normal and its inner diameter was slightly widened. The ratio of the right coronary artery to aortic root was 0.19 ± 0.02 (0.17–0.21); fibrosis of mitral tendinous cord and papillary muscle; mitral valve prolapse with medium to large amount of reflux signals at the mitral valve orifice during systole; collateral circulation between the right coronary artery may be observed; the dysplasia left coronary artery may be observed if the image is clear enough but there was no connection between left coronary artery (LCA) and the left coronary ostium, and abnormal reflux signals in the dysplasia LCA may be observed by color doppler flow imaging (CDFI) (Figure 3).

**Figure 4:**
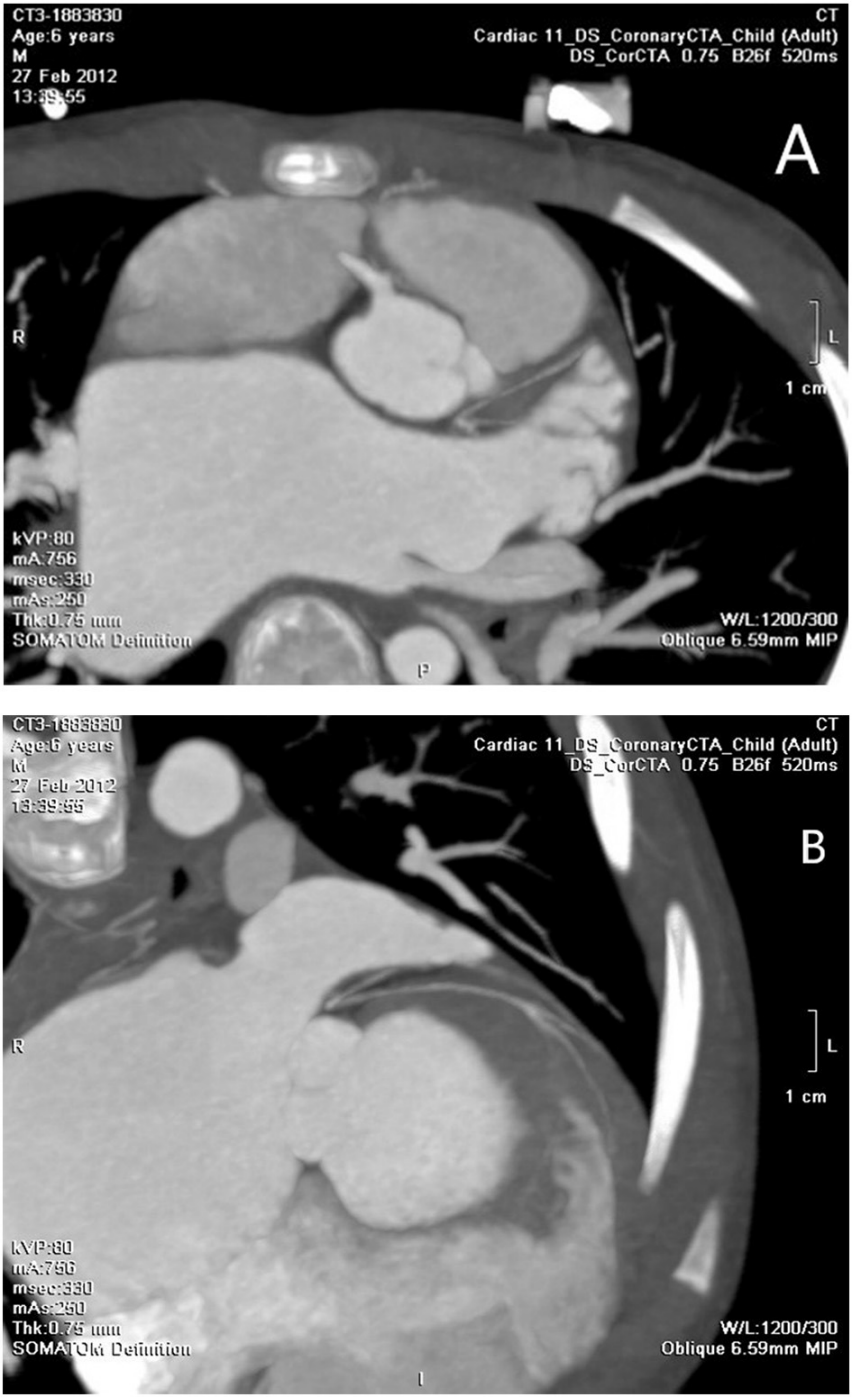
**(A)** Cardiac CTA: No left coronary artery opening in the left coronary sinus. **(B)** Cardiac CTA: hypoplasia of left anterior descending coronary artery and left circumflex coronary artery.

A number of 5 patients underwent cardiac CTA examinations. LMCAA was confirmed in 2 of the 5 children. Suspicious left coronary artery abnormalities were reported in 2 children, and no abnormalities were reported in 1 child. The main signs of cardiac CTA with LMCAA are the enlargement of left atrium and ventricle, normal opening of right coronary artery in right sinus and unobstructed coronary artery lumen, and proximal atresia of left main coronary artery. Furthermore, the left anterior descending coronary artery and the left circumflex coronary artery develop intermittently, with light development and thin lumen (Figure 4).

**Figure 5:**
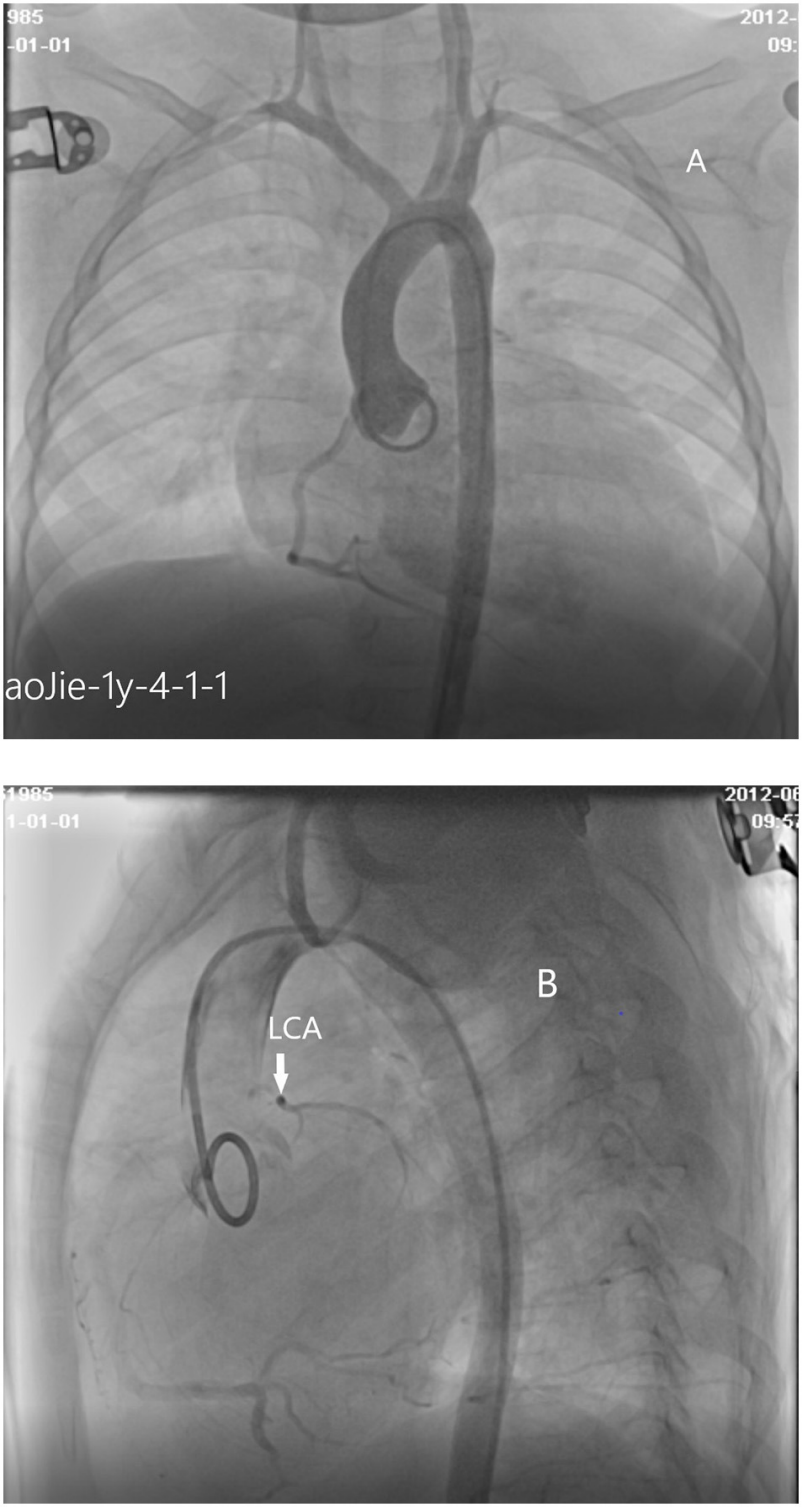
**(A)** Cardiac angiography: No left coronary artery opening is found in the left coronary sinus by aortography. **(B)** Cardiac angiography:atresia of left coronary ostium and hypoplasia of main stem of left coronary artery.

A number of 8 patients underwent coronary angiography. Coronary angiography can clearly show the shape and developmental sequence of coronary arteries. Aortic root angiography shows that the origin and course of the right coronary artery are normal, the diameter is slightly thicker, and the left coronary sinus is a blind end. Selective right coronary angiography shows that the branches of the left coronary artery are inversely perfused through the lateral branches of the right coronary artery, and the hypoplasia left coronary artery was finally developed and showed a blind end. No contrast agent developed in the pulmonary artery sequentially which means that there is no connection between LMCAA and pulmonary artery (PA) that often seen in the angiography of anomalous left coronary artery from the pulmonary artery (ALCAPA) (Figure 5). The right cardiac catheterization data showed the normal range of pulmonary artery pressure in these 8 children. The calculation of Qp/Qs did not indicate left to right shunt.

## Treatment and Follow-Up

The children with congenital left main coronary artery atresia (CLMCAA) mainly showed the symptoms as myocardial ischemia, mitral regurgitation, and cardiac insufficiency. After diagnosis, the heart failure treatment started, such as digoxin, diuretics, and captopril. Follow-up time was 1–8 years. The symptoms were stable in 8 patients. One child who was diagnosed at 7 months died of heart failure at the age of 9 years. A number of 3 children underwent coronary angioplasty and mitral valvuloplasty after the definite diagnosis. They were followed up for 11–25 months, and the results were good until now. No symptoms happened in the cases, blood flow at the opening of the left coronary artery was unobstructed, and the cardiac function was normal. The electrocardiogram showed normal results. Due to the high surgical difficulty of LMCAA, the number of cases of operation in China was small. Some of the children's families could not accept the risk of surgery, so the remaining children continued to take oral medication, and their symptoms were stable at present. The clinical condition, examination, and diagnosis results of 12 children are shown in Tables 1, 2.

**Table 1:** Clinical data of atresia of the left main coronary artery in 12 children.

**Case**	**Gender**	**Age of onset**	**Age of diagnosis**	**Weight (kg)**	**Preliminary diagnosis**	**Symptoms**	**Treatment and follow-up outcome**
1	M	2 m	7 m	6.5	LVNC	Recurrent Respiratory infection, Heart murmur Feeding difficulty	Followed up for 8 years with medicine treatment, and died at the age of 9
2	F	1 y	6 y	24	EFE	Pneumonia, Recurrent syncope	Followed up for 5 years, recurrent syncope, improved after 3 years with medicine treatment
3	M	1 y	5 y	19.2	Mitral regurgitation (severe)	Respiratory infection, Heart murmur	**Followed up for 3 years with medicine treatment. Left ventricular aneurysm occurred at the age of 6 years and received surgical treatment at 8 years old**
4	F	2 y	5 y	15	Mitral regurgitation (severe)	Respiratory infection, Cardiac enlargement	Followed up for 7 years, with medicine treatment, stable at present
5	M	5 m	1 y 5 m	11.3	Mitral regurgitation (moderate)	Respiratory infection, Cardiac enlargement	Followed up for 7 years, with medicine treatment, stable at present
6	F	2 y	2 y 11 m	12.2	Abnormal origin of left coronary artery?	Pneumonia, Cardiac enlargement	Followed up for 4 years, with medicine treatment, stable at present
7	M	5 m	7 m	7	LMCAA	Pneumonia, Heart murmur	Followed up for 4 years, with medicine treatment, stable at present
8	F	7 m	10 m	7.3	Mitral regurgitation (severe)	Heart murmur	Followed up for 4 years, with medicine treatment, stable at present
9	M	3 m	1 y	10	Mitral valve prolapse and insufficiency	Respiratory infection, Heart murmur	Followed up for 2 years, and received surgical treatment at the age of 3, recovered well and continue to take medicine
10	M	1 y 6 m	3 y 6 m	13	DCM	Feeding difficulty, Cardiac enlargement	Followed up for 2 years, with medicine treatment, stable at present
11	M	6 m	11 m	10	Mitral regurgitation	Heart murmur	Followed up for 1 years, with medicine treatment, stable at present
12	M	7 m	8 m	8	Mitral regurgitation (severe)	Feeding difficulty, Cardiac enlargement	Followed up for 2 years, and received surgical treatment at the age of 2, recovered well and continue to take medicine

**Table 2:** Echocardiogram, ECG, and X-ray cardiogram in 12 children.

**Case**	**Gender**	**Age of diagnosis**	**ECG**	**CXR**	**Echocardiogram**					
					**LVEDD *Z*-Value**	**LVEF (%)**	**Mitral regurgitation**	**RCA/AO**	**Collateral formation**	**Diagnosis**
1	M	7 m	Abnormal Q waves in I, AVL, V3, V5 leads, extensive ST-T depression	0.61	7.44	46	Severe	0.17	Little	Non-compaction cardiomyopathy
2	F	6 y	No obvious abnormality	0.52	2.05	62	Moderate	0.17	Little	Endocardial fibroelastosis
3	M	5 y	Abnormal Q waves in I, AVL leads	0.53	4.05	64	Severe	0.17	Little	Mitral regurgitation
4	F	5 y	Abnormal Q waves in V5,V6 leads, ST segment depression in v4–v6 leads	0.51	2.06	74	Severe	0.21	Abundant	Mitral regurgitation
5	M	1 y 5 m	abnormal Q waves in I, AVL,V4–V6 leads	0.68	4.86	50	Moderate	0.20	Little	Mitral regurgitation
6	F	2 y 11 m	No obvious abnormality	0.57	4.43	61	Severe	0.19	Medium	Anomalous origin of left coronary artery
7	M	7 m	abnormal Q waves in I, AVL leads	0.58	7.05	59	Severe	0.21	Medium	Congenital atresia of the left main coronary artery
8	F	10 m	No obvious abnormality	0.62	7.13	65	Severe	0.20	Little	Anomalous origin of left coronary artery
9	M	1 y	No obvious abnormality	0.67	4.49	64	Severe	0.18	Medium	Mitral regurgitation
10	M	3 y 6 m	Abnormal Q waves in I, AVL leads	0.53	4.41	40	Moderate	0.17	Little	Dilated cardiomyopathy
11	M	11 m	No obvious abnormality	0.65	4.02	73	Severe	0.21	Medium	Mitral regurgitation
12	M	8 m	Abnormal Q waves in I, AVL leads	0.64	3.57	63	Severe	0.19	Medium	Mitral regurgitation

## Discussion

There are various classification methods for congenital coronary abnormalities. Angelini (3) proposed a classification based on the anatomical features of abnormal coronary artery development, which includes anomalies of origination, course, and termination. CLMCAA is classified as the type of abnormal coronary course. At present, the pathogenesis of CLMCAA is not clear. The possible mechanisms include congenital loss or displacement of left coronary artery primordium, failure of left coronary artery opening, an extension of aortic mediator fiber to left coronary artery opening, or early embryonic coronary artery obstruction (4).

Upon to the reports (5), the onset age of LMCAA varies from 2 months to 85 years and the clinical manifestations of different ages are also different. Infants mostly show the symptoms as chronic heart failure, such as dyspnea, recurrent pneumonia, feeding difficulty, and so on Syncope and chest pain are the main symptoms in elder children and adults. In this group, the cases were all young and had onset early in infancy. The main complaints were cardiac enlargement, cardiac insufficiency, cardiac murmur, and syncope. Owing to the lack of specificity in the clinical manifestations and rare morbidity of the disease, LMCAA has a higher rate of false initial diagnosis. The patients had been misdiagnosed as valvular disease, endocardial fibroelastosis, left ventricular non-compaction, dilated cardiomyopathy, and anomalous origin of the left coronary artery from the pulmonary. The misdiagnosis rate of an initial visit of our patients was as high as 91.7% (11/12). The reason is mainly because LMCAA is extremely rare, and always confused with other diseases of cardiac disfunction such as endocardial fibrolastosis, dilated cardiomyopathy, ALCAPA, valvular diseases et al, and doctors lacked experience. The first case we diagnosed was suspected as abnormal origin of left coronary artery from echocardiogram, but different from ALCAPA, and the diagnosis was at last done by aorta root angiography.

The combination of clinical manifestations and imaging is the basis for the diagnosis of LMCAA. Chest X-ray shows enlarged heart shadow and pulmonary congestion in 8/12 cases, which is non-specific but can indirectly suggest heart disfunction in children. Electrocardiogram (EKG) is commonly and easily used in clinic but sometimes is ignored. In our groups,7/12 patients showed abnormal deep Q waves in leads I, AVL, and V4–V6 in EKG, which was also often seen in ALCAPA (6) and suggest severe myocardial ischemia.

Echocardiogram has good repeatability and is valuable for the diagnosis of LMCAA (7) although it is very difficult to distinguish the origin of coronary especially in infants. It can provide evidence for whether further examination such as angiography or cardiac CTA is needed (8). Some details can be found in echocardiogram, which suggests the abnormality coronary origin. In our group, we noticed that sign of echo enhancement of mitral chordae tendineae and papillary muscles which suggest severe myocardial ischemia might distinguish LMCAA from other non-coronary diseases. Right coronary artery widened slightly and the opening was normal. Various degrees of coronary collateral arteries are the important signs of abnormality origin of coronary artery, which can distinguish with the diseases without coronary abnormality such as EFE and cardiomyopathy. If possible, the origin of coronary artery should be detected carefully, hypoplasia LCA with atresia ostium may be demonstrated in 2D echocardiogram, and the abnormal reflux blood signals in hypoplasia main left coronary attery (MLCA) showing in CDFI also suggest the possibility of LMCAA.

For patients with suspected coronary artery abnormalities, further examination such as CTA or coronary angiography should be recommended. With recent improvements in coronary artery visibility on CT in children, the diagnostic accuracy and the resultant clinical utility of coronary CTA are noticeably increasing in children (9). It had been reported (10) that cardiac CTA was a very effective and non-invasive examination for the diagnosis of this disease, which could clearly show the opening and course of the left and right coronary arteries, as well as the widening degree of the right coronary artery. In our group, 5 cases underwent cardiac CTA, and 2 elder cases had clear CT imaging, which showed that the proximal segment of the left coronary artery was blind and dysplastic. However, cardiac CTA examination had limitations on infant coronary artery examination because breath shortness in some patients and rapid heart rate may affect image quality to a certain extent. Aortic root angiography and selective angiography are the golden indexes for the diagnosis of LMCAA. A number of eight children in our group underwent angiography. Angiography could clearly show the course and lumen shape of coronary artery (11). The developing sequence is the right coronary artery develops first, and then collateral artery, at last hypoplastic left main coronary artery, and the atresia ostium can be shown clearly. No development of pulmonary artery was the important sign to distinguish with ALCAPA. Selective right coronary angiography was required for elder patients with an unclear display.

The differential diagnosis of LMCAA includes single coronary artery, ALCAPA, and left coronary artery occlusion caused by other reasons (12). The anatomical change of LMCAA is that the aortic sinus has only one single right coronary artery opening, but its hemodynamics is different from that of single coronary artery. In the single coronary malformation, the blood supply direction is normal, branching from the main coronary artery to the small coronary arteries, while in the LMCAA, the blood from the right coronary artery flows back to the left coronary artery through one or more collateral branches. Generally, the collateral circulation from the right coronary to the left coronary cannot meet the metabolic needs of the heart, so almost all patients will suffer from myocardial ischemia (13). The clinical manifestations of ALCAPA are similar to those of LMCAA. The change of cardiac enlargement, decreased left ventricular ejection fraction, endocardial fibroelastosis, mitral insufficiency, and special electrocardiogram (ECG) changes are all caused by abnormal origin of LCA. Due to the abnormal left coronary artery originated from the pulmonary artery, the low pulmonary artery pressure results in the relatively serious right coronary artery steal, and the widening of the right coronary artery is more obvious. Yu et al. (14) summarized 30 children with infantile ALCAPA whose left ventricle was significantly enlarged and the RCA/AO ratio was >0.12. Different degrees of mitral regurgitation accounted for 60% of the patients. Different from ALCAPA patients, the widening of RCA was not obvious in our 12 children with LMCAA, and the RCA/AO ratio was 0.17–0.21 (0.19 ± 0.02). The Sign of coronary blood flow into pulmonary artery in CDFI of echocardiogram is helpful to distinguish the two diseases, and aortic root angiography or selective right coronary angiography can make a definite diagnosis. LMCAA should be distinguished from complete occlusion of the left main coronary artery second to other diseases (such as Kawasaki disease, Takayasu arteritis, and so on). In these cases, the main trunk is occluded but the anatomical morphology and inner diameter are normal.

We first reported this disease in China, and so far, it has been reported the most in a single center (12). The patients in our group were mainly treated with oral medicine to maintain cardiac function. The number of 1 patient died because of heart failure. Only 3 children underwent cardiac surgery mainly because of the difficulty in operation and parent's hesitation of risk of operation. Some researchers believed that surgical treatment was not needed on asymptomatic children with abundant collateral circulation and normal heart function. However, sudden death can occur at any age, and the severity of symptoms may be related to the development of collateral between right coronary artery and left coronary artery (2). Most researchers believed that due to the poor prognosis of the disease, the diagnosis itself of LMCAA was the surgical indication, and the purpose of the operation is to establish a normal dual coronary blood supply system.

Alsalehi et al. (2) reviewed 50 children diagnosed with LMCAA and strongly recommended immediate coronary revascularization due to its high incidence of heart failure and sudden death. Surgical procedures included coronary artery bypass grafting and coronary angioplasty. The choice of surgical procedure was mainly depended on the length from the orifice of the left coronary artery in the aortic sinus to the site of atresia. In the recent years, it was considered that coronary angioplasty was superior to coronary artery bypass grafting. Coronary angioplasty was referred to the reconstruction of the left main artery with autologous pericardial patch or arterial wall. After the successful operation, the left coronary artery was available to be redeveloped, which could provide more adequate blood flow for the blood supply area of the left coronary artery, while bypass grafting could only supply the area near the transplanted vessel. In 1972, Mullins et al. (15) reported the first case of great saphenous vein transplantation for the treatment of the disease. In 1992, Kitamura (16) reported that a 7-year-old girl was diagnosed as LMCAA complicated with severe mitral regurgitation and underwent coronary artery bypass grafting and mitral valve repair. Isotope myocardial perfusion scan after the surgery indicated significant improvement in myocardial blood supply and mild mitral valve regurgitation. In the follow-up of nearly 30 years after the operation, it was found that the patient's mitral regurgitation was aggravated, but no surgery is required. In 2017, Fujita et al. (17) reported that a 13-year-old child with syncope onset underwent bypass grafting and did not experience subsequent syncope. Albadi et al. (18) reported a case of left main coronary artery reconstruction with the autologous pericardium, which connects the anterior descending branch and circumflex branch with the aortic root. Then, 3 years after the surgery, CTA showed that the left main trunk was unobstructed and there was no calcification. In this group, three children underwent coronary angioplasty and mitral valvuloplasty. They all underwent coronary angioplasty using the anterior wall of the main pulmonary artery, and the surgical effect was good. At present, they were being followed up. After the operation, they continued to take oral diuretics and medicine to improve myocardial remodeling.

In conclusion, LMCAA was rare in clinic, but with the improvement of diagnostic experience and understanding of the disease, more and more coronary malformations would be found. Patients with cardiac insufficiency, a large amount of regurgitation in mitral valve, and pathological Q wave found by echocardiogram should be vigilant about the diagnosis of left coronary artery disease. Furthermore, cardiac CT and coronary angiography should be considered when necessary (19). Medical drug treatment could effectively improve and keep cardiac function, but surgery was the fundamental treatment for the disease.

## Data Availability Statement

The original contributions presented in the study are included in the article/supplementary material, further inquiries can be directed to the corresponding author/s.

## Author Contributions

XJ led the overall study, contributed to the data collection and interpretation, and wrote the manuscript. YX contributed to the data collection, data analysis, and manuscript edits. WY contributed to the data interpretation and manuscript edits. All authors read, contributed to the research design, and approved the final manuscript.

## Conflict of Interest

The authors declare that the research was conducted in the absence of any commercial or financial relationships that could be construed as a potential conflict of interest.

## Publisher's Note

All claims expressed in this article are solely those of the authors and do not necessarily represent those of their affiliated organizations, or those of the publisher, the editors and the reviewers. Any product that may be evaluated in this article, or claim that may be made by its manufacturer, is not guaranteed or endorsed by the publisher.

## References

[1] Yildiz A Okcun B Peker T Arslan C Olcay A Bulent Vatan M. Prevalence of coronary artery anomalies in 12,457 adult patients who underwent coronary angiography. Clin Cardiol. (2010) 33:E60–4. 10.1002/clc.20588 21184546 PMC6653639

[2] Alsalehi M Jeewa A Wan A Contreras J Yoo SJ Laks JA. A case series of left main coronary artery ostial atresia and a review of the literature. Congenit Heart Dis. (2019) 14:901–23. 10.1111/chd.12842 31532081

[3] Angelini P. Coronary artery anomalies:an entity in search of an identity. Circulation. (2007) 115:1206–305. 10.1161/CIRCULATIONAHA.106.618082 17353457

[4] Unzué L García E Parra FJ Palomo J Friera LF Solís J. Congenital atresia of the left main coronary artery in an adult: a rare anomaly with an unfavorable prognosis. Review of the literature. Cardiovasc Revasc Med. (2015) 16:498–502. 10.1016/j.carrev.2015.08.006 26382034

[5] Xiao Y Han L Jin M Ding W. Congenital atresia of left main coronary artery in 4 children: case report and literature review. Zhonghua Er Ke Za Zhi. (2014) 52:383–6. 10.3760/cma.j.issn.0578-1310.2014.05.014 24969939

[6] Beyaz MO Çoban S Ulukan MÖ Dogan MS Erol C Saritaş T. Current strategies for the management of anomalous origin of coronary arteries from the pulmonary artery. Heart Surg Forum. (2021) 24:E065–71. 10.1532/hsf.3421 33635248

[7] Weigand JD Gowda S Lorber R Madan N. Echocardiographic diagnosis of left main coronary artery atresia. World J Pediatr Cong Heart Surg. (2017) 8:101–2. 10.1177/2150135116664702 28033085

[8] Luo X Li Z. Congenital atresia of the left main coronary artery: imaging feature in children. Echocardiogram. (2019) 36:1941–3. 10.1111/echo.14469 31487070

[9] Goo HW. Coronary artery imaging in children. Korean J Radiol. (2015) 16:239–50. 10.3348/kjr.2015.16.2.239 25741188 PMC4347262

[10] Al-Umairi RS Al-Kindi F Al-Tai S. Prevalence and spectrum of coronary anomalies detected on coronary computed tomography angiography: a single centre experience in Oman. Int J Cardiol Sultan Qaboos Univer Med J. (2019) 19:e108–13. 10.18295/squmj.2019.19.02.005 31538008 PMC6736262

[11] Raju V Hebbale RC Muniswamy CS Sivanna U. True congenital atresia of the left main coronary ostium: delayed presentation. Asian Cardiovasc Thorac Ann. (2018) 26:54–6. 10.1177/0218492317739473 29058975

[12] Xiao Y Jin M Han L Ding W Zheng J Sun C. Two congenital coronary abnormalities affecting heart function: anomalous origin of the left coronary artery from the pulmonary artery and congenital left main coronary artery atresia. Chin Med J. (2014) 127:3724–31. 10.3760/cma.j.issn.0366-6999.20133224 25382327

[13] D'Souza TF Samuel BP Vettukattil JJ Haw MP. Surgical treatment of neonate with congenital left main coronary artery atresia. Ann Thorac Surg. (2016) 101:352–5. 10.1016/j.athoracsur.2014.12.104 26694277

[14] Yu Y Wang QS Wang XF Sun J Yu LW Ding M. Diagnostic value of echocardiography on detecting the various types of anomalous origin of the left coronary artery from the pulmonary artery. J Thorac Dis. (2020) 12:319–28. 10.21037/jtd.2020.01.28 32274098 PMC7139093

[15] Mullins CE El-Said G McNamara DG Cooley DA Treistman B Garcia E. Atresia of the left coronary artery ostici: repair by saphenous vein graft. Circulation. (1972) 46:989–94. 10.1161/01.CIR.46.5.989 4538780

[16] Kitamura S Kawachi K Nishii T Taniguchi S Inoue K Mizuguchi K. Internal thoracic artery grafting for congenital coronary malformations. Ann Thorac Surg. (1992) 53:513–6. 10.1016/0003-4975(92)90283-A 1540074

[17] Fujita S Sato A Nagata Y Usuda K Murata A Hatasaki K. Congenital left main coronary artery atresia presenting as syncope and generalized seizure during exercise in a 13-year-old boy. J Cardiol Cases. (2017) 16:126–30. 10.1016/j.jccase.2017.06.004 30279815 PMC6149280

[18] Albadi W Martin A Kreitmann B Roubertie F. Anatomic repair of a left coronary artery main stem atresia. Can J Cardiol. (2019) 35:1419.e5–7. 10.1016/j.cjca.2019.06.011 31601416

[19] Tian M Wang X Gao H Wang L Hu S. Left main coronary artery atresia with concomitant mitral regurgitation in an adult: a case report. Medicine. (2018) 97:41. 10.1097/MD.0000000000012367 30313032 PMC6203469

